# The cytosolic N-terminus of CD317/tetherin is a membrane microdomain exclusion motif

**DOI:** 10.1242/bio.20135793

**Published:** 2013-10-15

**Authors:** Peter G. Billcliff, Oforiwa A. Gorleku, Luke H. Chamberlain, George Banting

**Affiliations:** 1 School of Biochemistry, University of Bristol, Bristol BS8 1TD, UK; 2 Strathclyde Institute of Pharmacy and Biomedical Sciences, University of Strathclyde, Glasgow G4 0RE, UK; * Present address: Faculty of Life Sciences, University of Manchester, Manchester M13 9PL, UK

**Keywords:** tetherin, Membrane microdomain, Lipid raft, Palmitoylation

## Abstract

The integral membrane protein CD317/tetherin has been associated with a plethora of biological processes, including restriction of enveloped virus release, regulation of B cell growth, and organisation of membrane microdomains. CD317 possesses both a conventional transmembrane (TM) domain and a glycophosphatidylinositol (GPI) anchor. We confirm that the GPI anchor is essential for CD317 to associate with membrane microdomains, and that the TM domain of CD44 is unable to rescue proper microdomain association of a ΔGPI-CD317 construct. Additionally, we demonstrate that the cytosolic amino terminal region of CD317 can function as a ‘microdomain-excluding’ motif, when heterologously expressed as part of a reporter construct. Finally, we show that two recently described isoforms of CD317 do not differ in their affinity for membrane microdomains. Together, these data help further our understanding of the fundamental cell biology governing membrane microdomain association of CD317.

## Introduction

Membrane microdomains (aka lipid rafts), are regions of the plasma membrane (PM) enriched in cholesterol, sphingolipid, and specific proteins (Simons and Ikonen, 1997; Lingwood and Simons, 2010). Although their very existence has been questioned (Munro, 2003), there is extensive evidence that membrane microdomains are present in living cells, with these domains being small and, in some cases, highly dynamic (reviewed by Hancock, 2006; Lingwood and Simons, 2010; Simons and Gerl, 2010). Membrane microdomains have been implicated as being important for multiple biological processes, including membrane trafficking, cell signalling and exocytosis (reviewed by Simons and Toomre, 2000; Salaün et al., 2004; Gupta and DeFranco, 2007; Jury et al., 2007; Simons and Gerl, 2010). There is also much evidence implicating membrane microdomains as being critical for pathogen entry/egress, with, for example, both HIV and the influenza virus known to hijack them for virus budding (reviewed by Lafont and van der Goot, 2005; Lencer and Saslowsky, 2005; Goldston et al., 2012; Hogue et al., 2012). Additionally, membrane microdomains have been associated with the development of diseases, such as Alzheimer's and prion diseases (reviewed by Simons and Ehehalt, 2002; Michel and Bakovic, 2007; Hicks et al., 2012).

A significant amount of research has been performed to ascertain the features that influence the affinity of specific proteins for membrane microdomains. Glycophosphatidylinositol (GPI)-anchor-dependent sorting of proteins to membrane microdomains is well-characterised, with the presence of the long and saturated GPI motif conferring a strong affinity for membrane microdomains upon a protein (Suzuki et al., 2007; Lingwood and Simons, 2010). The addition, by palmitoylation, of a C16 palmitate moiety to a cysteine residue, is another lipid modification that can cause a protein to localise to membrane microdomains; indeed, a recent publication suggested that palmitoylation is required for appropriate partitioning of the majority of integral membrane microdomain proteins (Levental et al., 2010). Furthermore, membrane microdomain-associated integral membrane proteins have longer TM domains than non-membrane microdomain proteins (Alberts et al., 2008), and glycosylation may also cause preferential association with membrane microdomains, probably through microdomain-localised lectins binding to glycan moieties on proteins (Stechly et al., 2009). In addition, it has been proposed that membrane microdomain affinity is enhanced as a microdomain-localised protein oligomerises (Simons and Vaz, 2004).

CD317 (variously known as HM1.24 (Goto et al., 1994), Bst-2 (Ishikawa et al., 1995), tetherin (Neil et al., 2008)) is an integral membrane protein with an unusual topology. The protein consists of a short amino terminal cytosolic tail, a conventional transmembrane (TM) domain, an extracellular region containing an extended coiled-coil that causes the protein to assemble into dimers (Hinz et al., 2010; Schubert et al., 2010; Yang et al., 2010; Swiecki et al., 2011), and a C-terminal GPI anchor (Kupzig et al., 2003; Elortza et al., 2006). CD317 has been implicated as playing a role in a variety of cellular processes, including regulation of B cell growth and development (Goto et al., 1994; Ishikawa et al., 1995; Ohtomo et al., 1999), cell adhesion (Yoo et al., 2011), and antiviral response (Neil et al., 2008; Van Damme et al., 2008). This latter role, in which CD317 restricts virus release by acting to directly tether enveloped viruses at the surface of infected cells, inspired the protein to be designated ‘tetherin’ (Neil et al., 2008). Although CD317 was initially identified as an inhibitor of HIV virion release (Neil et al., 2008; Van Damme et al., 2008), it has since been shown to restrict the release of a diverse range of enveloped viruses (reviewed by Le Tortorec et al., 2011).

Consistent with the presence of a GPI addition motif at the carboxyl terminus of CD317, the protein is localised to membrane microdomains. Biochemical studies, employing the classical method of lysing cells in the detergent Triton X-100 prior to separation of lysates by density gradient ultracentrifugation, have demonstrated that CD317 resides in detergent-resistant membranes (DRMs) (Kupzig et al., 2003; Masuyama et al., 2009; Rollason et al., 2009), which bear many of the characteristics of membrane microdomains. Additionally, super-resolution microscopy has shown that CD317 resides in largely immobile clusters of 70 to 150 nm in diameter in the PM (Lehmann et al., 2011; Hammonds et al., 2012), consistent with localisation to membrane microdomains. Evidence from analysis of DRMs (Kupzig et al., 2003) and super-resolution imaging (Lehmann et al., 2011) suggests that the presence of the GPI anchor is critical for membrane microdomain localisation of CD317. However, work performed by Andrew et al. suggested that, at least in some cell types, the carboxyl terminal region of CD317 is able to function as a second TM domain, as opposed to a GPI anchor addition sequence (Andrew et al., 2011).

We have previously demonstrated that CD317 is an organiser of membrane microdomains (Billcliff et al., 2013). In the work presented here, we sought to determine if a TM domain from a heterologous protein can functionally replace the GPI anchor in CD317, at least with regards to localising the protein to membrane microdomains. We also present work, related to this, which led us to conclude that the amino terminal region of CD317 is able to act as a membrane microdomain-excluding motif. Finally, we assessed the membrane microdomain/non-membrane microdomain localisation of two recently described (Cocka and Bates, 2012) isoforms of CD317 that bear cytosolic domains of differing lengths.

## Results

### The GPI anchor addition motif is critical for membrane microdomain localisation of CD317

**Fig. 1.**
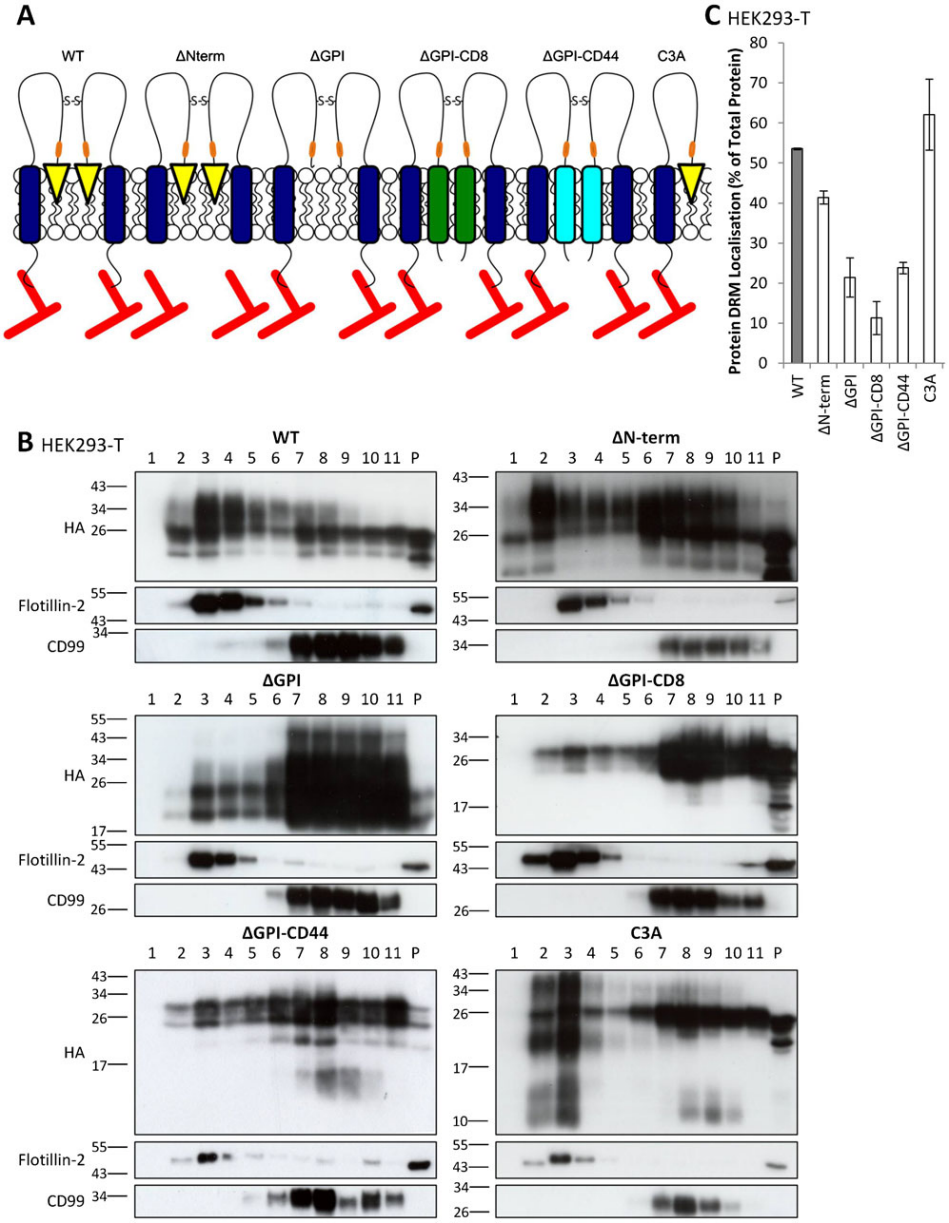
The CD44 transmembrane domain does not restore membrane microdomain association of a CD317 construct lacking the GPI anchor. (A) Schematic of CD317 constructs. See main text for details. Rounded rectangles represent TM domains (dark blue – tetherin; green – CD8; cyan – CD44); yellow triangles, GPI anchor; orange bars, HA tag; red lines, actin cytoskeleton. (B) DRM/non DRM localisation of CD317 constructs. Immunoblot analysis of fractions from sucrose-density-gradient separation of HEK293-T cell lysates from cells transfected with the indicated CD317 constructs. Fractions were taken from the top of the gradient (i.e. fraction 1 is the most buoyant), and blots were probed with HA, flotillin-2 and CD99 antibodies, as indicated. (C) DRM localisation (protein in flotillin-2 positive fractions) was quantified by densitometry, with values given as protein localised to DRM as a percentage of total protein, ± s.e.m. (*n* = 2).

We have previously demonstrated that CD317 is important for membrane microdomain organisation in HeLa cells (Billcliff et al., 2013). In order to assess the relative importance of the various regions of CD317 in organising membrane microdomains, a panel of CD317-based constructs was designed (Billcliff et al., 2013) (Fig. 1A). Experiments utilising these constructs showed that the entire domain organisation of CD317 is critical for its capacity to organise membrane microdomains, with no single feature of CD317 being either sufficient or dispensable for this function (Billcliff et al., 2013).

The panel of CD317-based constructs consists of various deletion and substitution constructs that were generated in a HA-tagged and siRNA-resistant (SR) background (CD317-HA-SR) (Billcliff et al., 2013) (Fig. 1A). The panel consists of a construct that lacks the cytosolic domain of CD317 (ΔN-term) and one that lacks the GPI addition motif (ΔGPI). In addition, two constructs were generated in which the GPI anchor was replaced by a TM domain from another protein. Insertion of the CD8α TM domain, which would be predicted to lead to localisation to non-membrane microdomain regions of the plasma membrane (Pang et al., 2007), generated a ΔGPI-CD8 chimeric construct. Similarly, a ΔGPI-CD44 chimera was produced by insertion of the TM domain from CD44, a construct that was designed to be membrane microdomain-associated, given that the TM domain of CD44 confers a high affinity for membrane microdomains upon CD44 (Neame et al., 1995; Perschl et al., 1995). A final construct, designated C3A, was generated where protein dimerisation was severely impaired by mutation of all three extracellular cysteines to alanine residues. Thus, this group of CD317 constructs addressed the importance of the amino terminal cytosolic region; localisation to membrane microdomains (and, with the ΔGPI-CD8/CD44 constructs, the specific requirement for a GPI anchor, as opposed to an alternative membrane microdomain localisation motif); and disulphide bond mediated stabilisation of the coiled coil dimer, for CD317's putative role in membrane microdomain organisation.

Sucrose density gradient experiments on HeLa cells expressing the various CD317-HA-SR constructs demonstrated that membrane microdomain localisation of CD317 is not dependent upon protein dimerisation or the presence of the amino terminal cytosolic domain (Billcliff et al., 2013). However, as anticipated, the GPI anchor was critical for the partitioning of CD317 into membrane microdomains, and neither the CD8 nor the CD44 TM domains could functionally replace the GPI anchor, with regard to partitioning CD317 into membrane microdomains (Billcliff et al., 2013). These data contrast with work published by Andrew et al., which suggested that replacement of the GPI addition motif with the TM domains of either the membrane microdomain-associated protein CD40 or the non-membrane microdomain proteins CD45 or the TfR had no effect on the membrane microdomain localisation of CD317 (Andrew et al., 2011). A possible reason for the discrepancy between the findings of Andrew et al. and our previously published data is that Andrew et al. examined membrane microdomain localisation of the CD317 constructs in HEK293-T cells (Andrew et al., 2011), whilst our previous work (Billcliff et al., 2013) utilised HeLa cells. Given that CD317 is not expressed in unstimulated HEK293-T cells, but is expressed in HeLa cells (Neil et al., 2008), it is conceivable that hetero-dimerisation with endogenous CD317 could explain the differing results that have been seen. Consequently, the distribution of our panel of CD317-based constructs on sucrose gradients was examined in HEK293-T cells (Fig. 1B,C). No significant differences in protein partitioning into membrane microdomains were seen between HEK293-T cells and HeLa cells (Billcliff et al., 2013), with neither the CD8 nor the CD44 TM domains being able to restore membrane microdomain localisation of a CD317 construct that lacked the GPI addition sequence. The inability of the CD44 TM domain to confer a high affinity for membrane microdomains on ΔGPI-CD44 CD317-HA-SR led us to question whether additional regions of CD44 might be required in order to restore efficient membrane microdomain partitioning upon a CD317 protein that lacks the GPI addition motif.

### The addition of a cytosolic tail sequence causes aberrant processing of CD317-ΔGPI-CD44

**Fig. 2.**
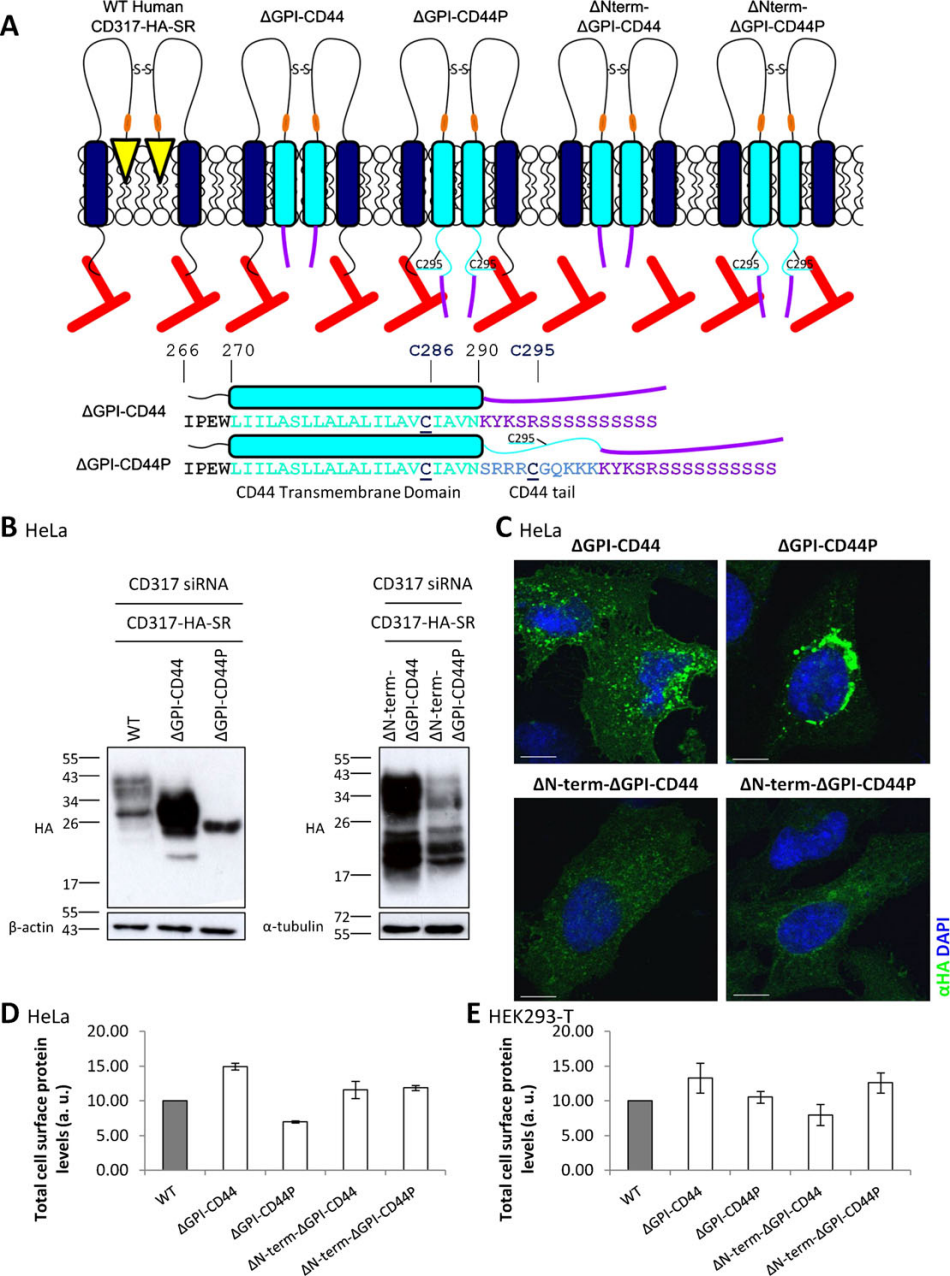
The addition of a cytosolic tail sequence causes aberrant processing of CD317-ΔGPI-CD44. (A) Schematic of CD317-ΔGPI-CD44 constructs. Rounded rectangles represent TM domains (dark blue – CD317; cyan – CD44); yellow triangles, GPI anchor; orange bars, HA tag; red lines, actin cytoskeleton. Beneath schematic, the sequences of the ΔGPI-CD44 and ΔGPI-CD44P constructs after the end of the CD317 region. CD44 TM domain in cyan; cytosolic tail in pale blue; palmitoylation sites in black, underlined. Numbers above sequences are residue numbers in CD44 transcript variant 4. Purple, motif to ensure constructs are delivered to the cell surface (Jackson et al., 1990). See main text, and Materials and Methods, for details. (B) Cell lysates from HeLa cells transiently co-transfected with siRNA targeting CD317 and the indicated CD317-HA-SR constructs were prepared in sample buffer, separated by SDS-PAGE and immunoblotted with antibodies specific to HA, β-actin or α-tubulin. Molecular mass markers are indicated in kilodaltons. (C) HeLa cells were co-transfected with siRNA targeting CD317 and the indicated CD317-HA-SR constructs and, 48 hours later, permeabilised by methanol fixation followed by immunofluorescence detection of the HA epitope to visualise whole cell localisation of CD317. (D,E) HeLa cells were co-transfected with siRNA targeting CD317 and the indicated CD317-HA-SR constructs and, 48 hours later, incubated with an antibody specific to HA to look at protein expression, before surface expression was monitored by flow cytometry in the PE channel. Quantification of CD317-HA-SR surface expression. Values indicate total fluorescence of transfected cells. (E) HEK293-T cells were transfected with the indicated CD317-HA-SR constructs and, 24 hours later, incubated with an anti-HA antibody before surface expression was monitored by flow cytometry in the PE channel. Values indicate total fluorescence of transfected cells. Data represent mean (± s.e.m.) from two independent experiments. Scale bars: 10 µm.

Research published in 2006 (Thankamony and Knudson, 2006), in which HEK293-T cells were stably transfected with a construct encoding CD44, demonstrated that, in addition to the TM domain of CD44, palmitoylation of specific residues is essential for it to localise to membrane microdomains (as assayed by lysis of samples in Triton X-100 followed by sucrose density gradient ultracentrifugation). Palmitoylation of two cysteine residues, C286 located within the TM domain, and C295 in the cytoplasmic tail at the carboxyl terminal end of the protein, was required for targeting of CD44 to membrane microdomains. Given that the CD317 ΔGPI-CD44 construct contains only the TM domain-localised C286 from CD44 (but not C295), it is possible that partitioning of the chimeric protein into membrane microdomains is impaired in the absence of the additional palmitoylation site provided by residue C295. To investigate this possibility, a second ΔGPI-CD44 chimeric construct was generated, identical to the original construct except for the addition of the ten amino acids of the cytoplasmic tail that are directly C-terminal to the TM domain (this includes C295). The new construct, designated ΔGPI-CD44P, is depicted in Fig. 2A, alongside ΔGPI-CD44. Two other, related, constructs, were also designed, in which the CD317 amino terminal region was removed from the two ΔGPI-CD44 constructs, to generate constructs designated ΔN-term-ΔGPI-CD44 and ΔN-term-ΔGPI-CD44P (Fig. 2A).

Surprisingly, the ΔGPI-CD44P protein, when expressed in HeLa cells, appeared as a single species with a molecular weight of about 26 kDa (or, as several species, where the predominant one was 26 kDa – Fig. 2B, left panel). The size of this species is consistent with the immature, unglycosylated form of CD317 (Andrew et al., 2009), suggesting the ΔGPI-CD44P construct is not being processed properly, and as a result is being trapped in the ER. Consistent with this theory, a high proportion of ΔGPI-CD44P-expressing HeLa cells displayed a bright perinuclear ring of immunofluorescence, indicative of localisation to the ER, when examined by immunofluorescence confocal microscopy (Fig. 2C). Additionally, flow cytometry analysis illustrated that that there was a greater than 50% reduction in total cell surface protein levels of ΔGPI-CD44P, relative to ΔGPI-CD44 (Fig. 2D). Together, these data demonstrate that, in HeLa cells, the addition of the cytoplasmic tail of CD44 to the ΔGPI-CD44 chimeric construct causes aberrant processing of the resultant construct. Although impairment in delivery of the ΔGPI-CD44P construct to the plasma membrane was also seen in HEK293-T cells, this effect was far subtler than that seen in HeLa cells, with only a 20% reduction in total cell surface protein levels seen (Fig. 2E). In accordance with this, immunoblot (supplementary material Fig. S1A) and immunofluorescence analysis of methanol-fixed cells (supplementary material Fig. S1B) illustrated that the ΔGPI-CD44P construct was processed far more efficiently in HEK293-T cells than in HeLa cells, with the majority of the protein being both properly glycosylated, and transported from the ER. In sharp contrast to the ΔGPI-CD44P construct, both the ΔN-term-ΔGPI-CD44 and ΔN-term-ΔGPI-CD44P proteins largely reflect the ΔGPI-CD44 construct with regards to appearance on immunoblots, localisation in methanol-fixed cells, and delivery to the PM. This was the case in both HeLa and HEK293-T cells (Fig. 2; supplementary material Fig. S1).

### Deletion of the CD317 N-terminus increases the partitioning of ΔGPI-CD44 into membrane microdomains in HEK293-T cells

**Fig. 3.**
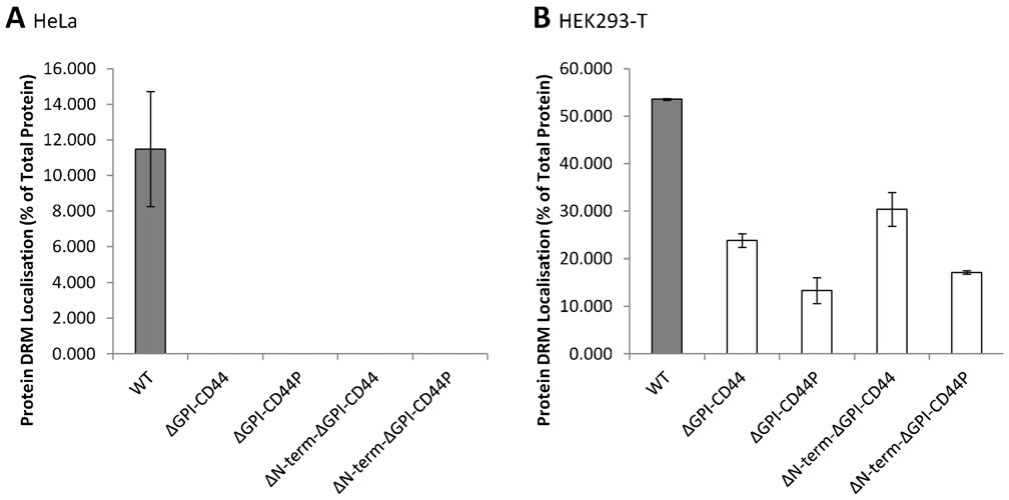
The deletion of the CD317 amino terminus leads to greater partitioning of CD317 ΔGPI-CD44 into HEK293-T cell membrane microdomains. DRM/non-DRM localisation of CD317 ΔGPI-CD44 constructs. HeLa (A) or HEK293-T cells (B) transfected with the indicated construct were lysed and DRMs prepared as described in Materials and Methods. DRM localisation (protein in flotillin-2 positive fractions) was quantified by densitometry, with values given as protein localised to DRMs as a percentage of total protein, ± s.e.m. (*n* = 2).

To establish whether addition of the second palmitoylation site of CD44 to the ΔGPI-CD44 construct, or the removal of the CD317 N-terminal cytosolic region from the ΔGPI-CD44 chimeric proteins, impacted upon their partitioning into membrane microdomains, HeLa and HEK293-T cells were transiently transfected with each ΔGPI-CD44 construct, and subsequently lysed in Triton X-100 before samples were separated by ultracentrifugation on a sucrose density gradient. Neither the addition of the cytosolic tail of CD44 to, nor the removal of the CD317 N-terminal region from, ΔGPI-CD44 affected the localisation of the ΔGPI-CD44 chimera to DRMs in HeLa cells (Fig. 3A; see supplementary material Fig. S2A for immunoblots). In HEK29-T cells, however, the results were more complicated (Fig. 3B; see supplementary material Fig. S2B for immunoblots). Whilst approximately two-fold less ΔGPI-CD44 was present in DRMs compared to WT CD317-HA-SR, even less (just over 10% of total protein, or under 25% relative to the WT protein) of the ΔGPI-CD44P construct was present in DRMs. With regards to the ΔN-term-ΔGPI-CD44 constructs, the removal of the cytosolic terminal domain of CD317 resulted in an increase in the proportion of both the ΔN-term-ΔGPI-CD44 constructs that were present in DRMs of HEK293-T cells. The increases were only small, resulting in about 30% more of both ΔN-term-ΔGPI-CD44 and ΔN-term-ΔGPI-CD44P partitioning into membrane microdomains, relative to ΔGPI-CD44 and ΔGPI-CD44P, respectively (Fig. 3). Thus, there was 50% of ΔN-term-ΔGPI-CD44 in DRMs relative to WT. However, the greater propensity of the ΔN-term-ΔGPI-CD44 constructs to reside in DRMs (as compared to the equivalent ΔGPI-CD44 constructs) indicates that, at least in HEK293-T cells, the removal of the N-terminal region of CD317 is enough to increase the DRM localisation of a CD317 construct in which the GPI anchor has been replaced by the TM domain of CD44.

### ΔGPI-CD44 CD317 constructs are not palmitoylated when expressed in HEK293-T cells

**Fig. 4.**
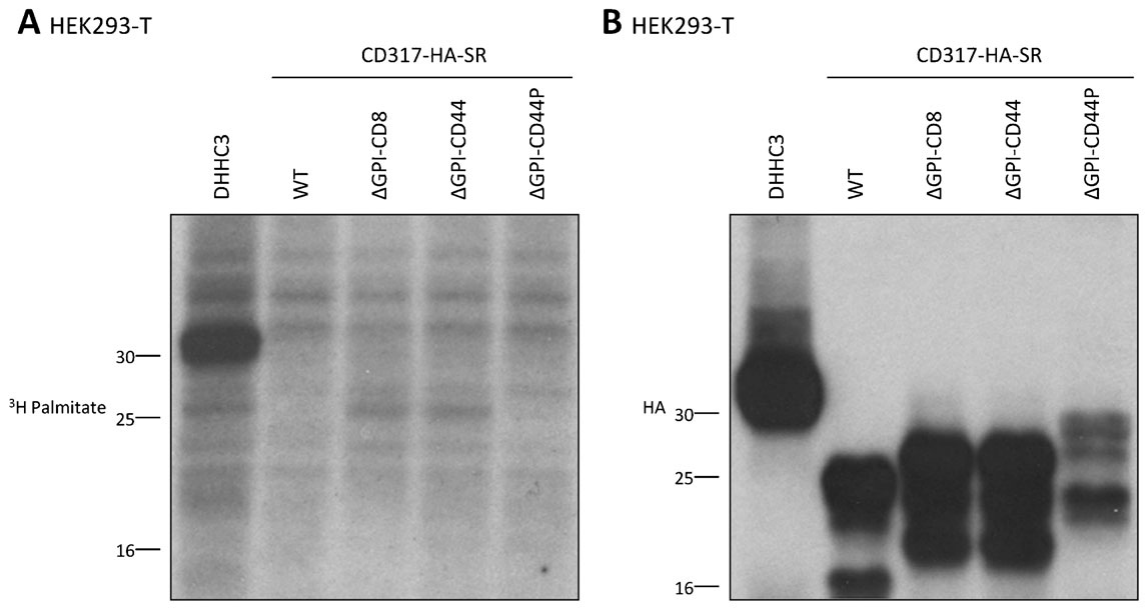
Palmitoylation of the ΔGPI-CD44 constructs is undetectable by 3H-palimtate labelling. HEK293-T cells were transfected with the indicated CD317-HA-SR construct, or DHHC3. Twenty-four hours later, cells were labelled for 3 hours with [3H]palmitate. Samples were separated by SDS-PAGE, and duplicate membranes transferred onto nitrocellulose for visualisation of the 3H signal with the aid of an intensifier screen (A), or for immunoblotting with an anti-HA antibody (B). Molecular mass markers are indicated in kilodaltons.

The greatly reduced partitioning into membrane microdomains in HEK293-T cells of both the ΔGPI-CD44 and ΔGPI-CD44P constructs, relative to WT CD317-HA-SR, suggests that the TM domain of CD44 is unable to functionally replace the GPI anchor of CD317, with respect to localising CD317 to membrane microdomains. This is particularly surprising in the case of the ΔGPI-CD44P construct, given that it contains both of the palmitoylation sites that are essential for membrane microdomain localisation of CD44 (Thankamony and Knudson, 2006). We therefore questioned whether the ΔGPI-CD44 and/or ΔGPI-CD44P constructs were efficiently palmitoylated. HEK293-T cells were transfected with each of the WT, ΔGPI-CD8, ΔGPI-CD44, or ΔGPI-CD44P CD317-HA-SR constructs, or an HA-tagged form of the palmitoyl acyl transferase DHHC3 (which is known to undergo auto-palmitoylation (Fukata et al., 2006)) as a positive control. Transfected cells were incubated with ^3^H-labelled palmitic acid, and incorporation of ^3^H-palmitate measured (as outlined in Materials and Methods). A clearly distinguishable ^3^H-labelled band was detected in lysates from cells expressing DHHC3 (Fig. 4A); this was of the same molecular weight as the predominant band visible in the corresponding immunoblot probed with an anti-HA antibody (Fig. 4B). However, only background ^3^H-labelled bands were detected in the samples from cells transfected with the CD317-HA-SR constructs, despite the constructs being expressed to a similar degree to the DHHC3 construct, as illustrated in the anti-HA immunoblot (Fig. 4B). Thus, the lack of any detectable palmitoylation of either of the ΔGPI-CD44 or ΔGPI-CD44P constructs provides one possible explanation for their reduced partitioning into membrane microdomains in HEK293-T cells, in comparison to the WT construct.

### The N-terminus of CD317 reduces the affinity of membrane proteins for membrane microdomains when heterologously expressed

**Fig. 5.**
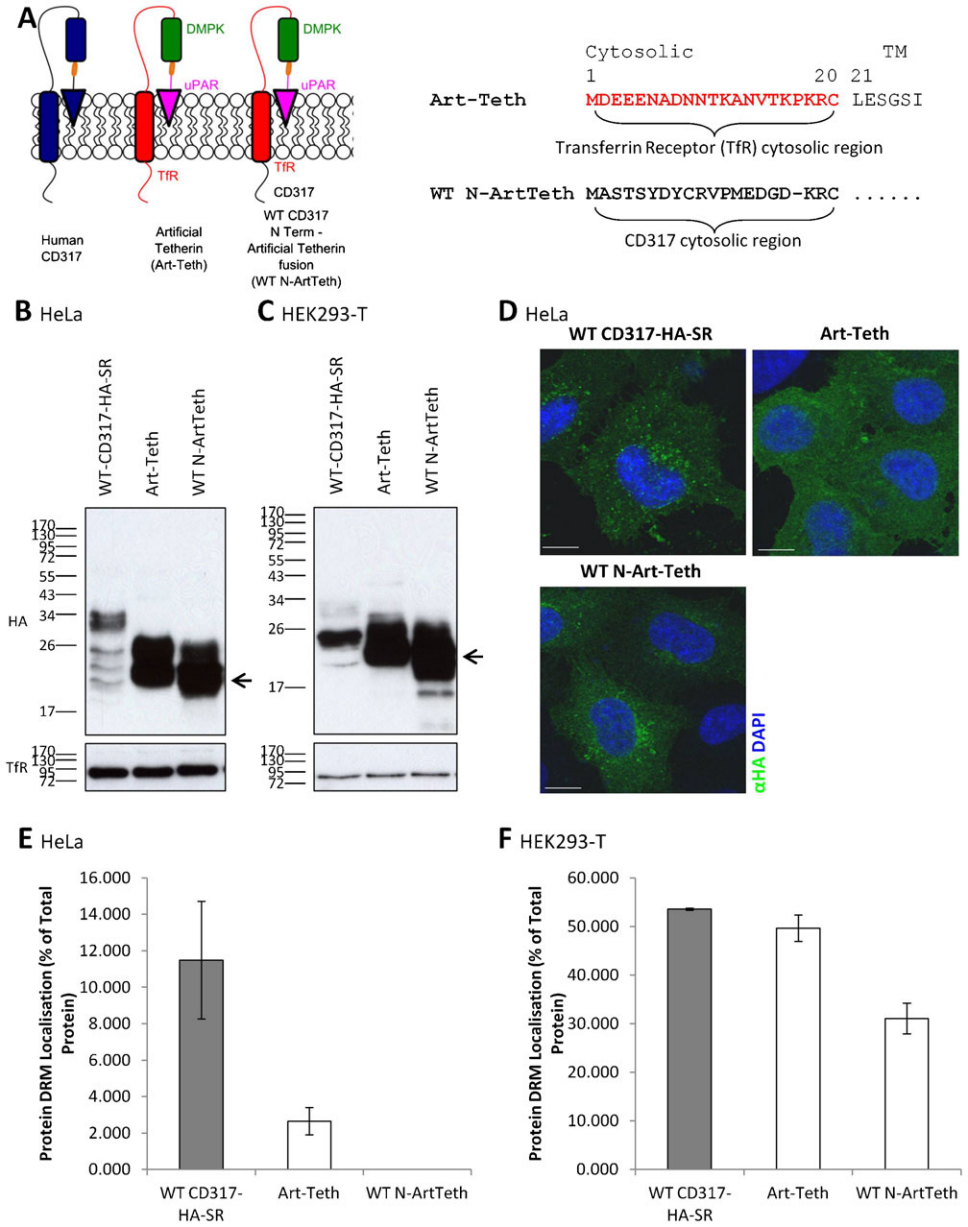
The cytosolic domain of CD317 reduces the affinity of artificial tetherin for membrane microdomains. (A) Left: schematic of artificial tetherin constructs. See main text, and Materials and Methods, for details. Rounded rectangles represent TM domains (dark blue – CD317; red – TfR); triangles, GPI anchor; orange bars, HA tag; smaller rounder rectangles coiled coil domains. Right: amino terminal cytosolic region of artificial tetherin constructs. (B,C) Cell lysates from HeLa cells (B) or HEK293-T cells (C) transiently transfected with the indicated artificial tetherin constructs, or WT CD317-HA-SR, were prepared in sample buffer, separated by SDS-PAGE and immunoblotted with antibodies specific to TfR or HA. Molecular mass markers are indicated in kilodaltons. Presumed degradation products of the Art-Teth constructs are indicated by arrowheads. (D) HeLa cells were transfected with the indicated artificial tetherin construct, or WT CD317-HA-SR, and, 48 hours later, permeabilised by methanol fixation followed by immunofluorescence detection of the HA epitope to visualise whole cell localisation of protein. (E,F) DRM/non-DRM localisation of artificial tetherin constructs. HeLa (E) or HEK293-T cells (F) transfected with the indicated construct were lysed and DRMs prepared as described in Materials and Methods. DRM localisation (protein in flotillin-2 positive fractions) was quantified by densitometry, with values given as protein localised to DRM as a percentage of total protein, ± s.e.m. (*n* = 2). Scale bars: 10 µm.

The increased affinity for membrane microdomain of both the ΔGPI-CD44 and ΔGPI-CD44P constructs for DRMs in HEK293-T cells following the removal of the cytosolic domain of CD317 (Fig. 3) raised the possibility that the CD317 cytosolic domain might reduce the affinity of membrane proteins for membrane microdomains when heterologously expressed. To test this theory, artificial tetherin (Art-Teth) was used as the host protein. Art-Teth is an entirely synthetic protein construct that consists of the N-terminus, TM domain and part of the extracellular stalk of the Transferrin receptor (TfR), the extracellular coiled coil region of dystrophia myotonica protein kinase (DMPK), and the GPI anchor addition motif of urokinase plasminogen activator receptor (uPAR) (Perez-Caballero et al., 2009). Thus, despite an absence of any sequence homology, Art-Teth assumes the same overall topology as CD317 (Fig. 5A). To address the importance of the cytosolic domain of CD317 for protein localisation to membrane microdomains, the cytosolic region of Art-Teth was replaced by the cytosolic region of CD317, to produce a chimeric construct that was designated WT N-ArtTeth (Fig. 5A). Both the Art-Teth and WT N-ArtTeth constructs were expressed and efficiently transported to the cell surface in transiently transfected HeLa and HEK293-T cells (Fig. 5B,C). It is notable that, whilst the Art-Teth protein fails to display a juxtanuclear pool of protein when expressed in HeLa cells, the WT N-ArtTeth chimera does do so, and is comparable to WT CD317-HA-SR in this respect (Fig. 5D).

HeLa and HEK293-T cells were transiently transfected with plasmids encoding either WT CD317-HA-SR or one of the artificial tetherin constructs, and subsequently lysed in Triton X-100 and samples separated by ultracentrifugation on sucrose density gradients. Art-Teth partitioned into DRMs in HeLa cells, although there was an approximately five-fold reduction in the proportion of protein that was DRM associated, in comparison to WT CD317-HA-SR (Fig. 5E; see supplementary material Fig. S3A for immunoblots). In contrast, the WT N-ArtTeth chimera was excluded entirely from DRMs of HeLa cells (Fig. 5E; supplementary material Fig. S3). Additionally, whilst the Art-Teth construct partitioned into DRMs in HEK293-T cells with similar efficiency to WT CD317-HA-SR, the affinity of the WT N-ArtTeth chimera for DRMs was significantly reduced, although not completely abrogated (Fig. 5F; see supplementary material Fig. S3B for immunoblots). These data indicate that the CD317 cytosolic domain reduces the affinity of Art-Teth for DRMs, and are consistent with the theory that the cytosolic domain of CD317 acts as a ‘membrane microdomain-exclusion’ motif.

### The long and short isoforms of CD317 do not differ in their affinity for membrane microdomains

**Fig. 6.**
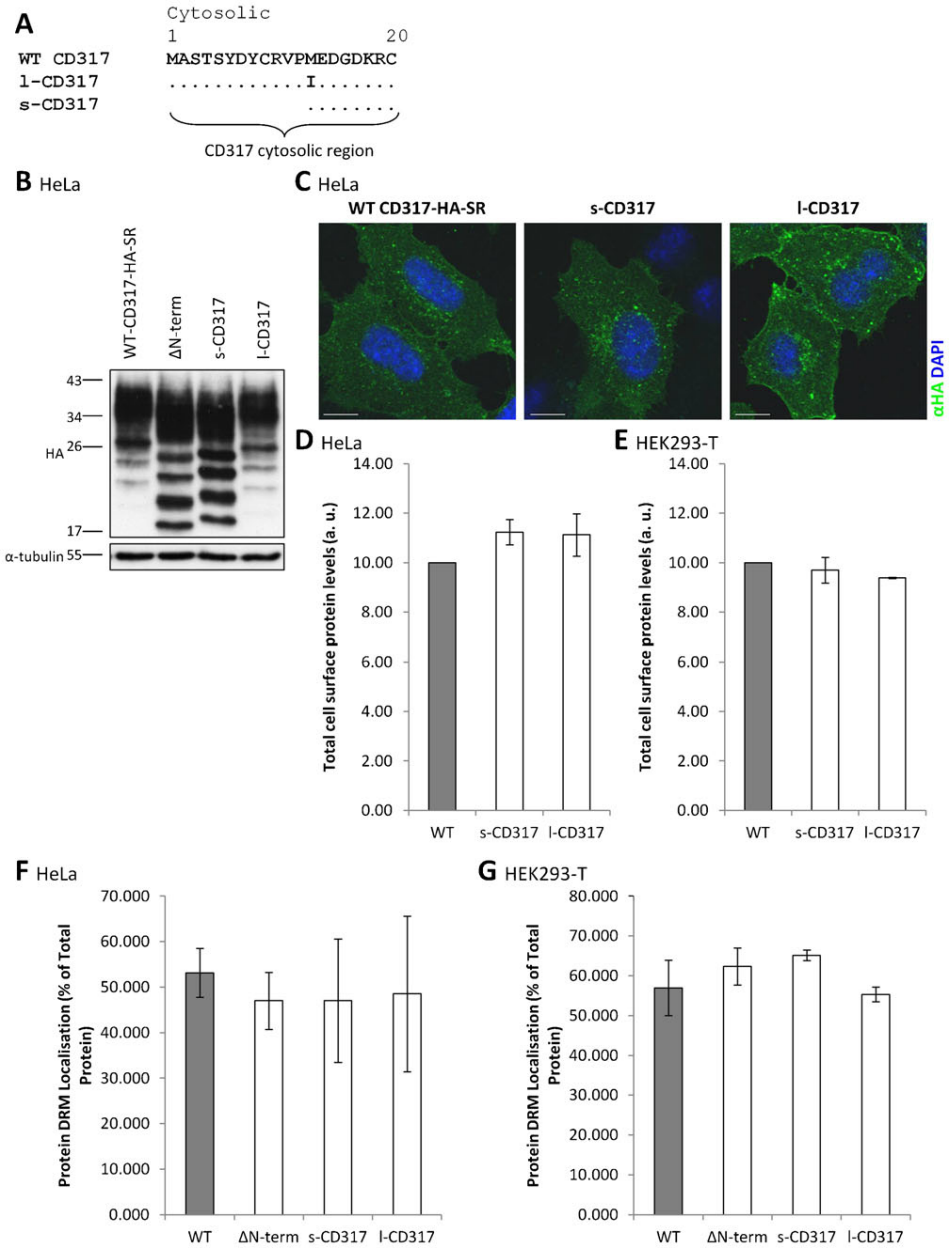
The long and short isoforms of CD317 do not differ in their affinity for membrane microdomains. (A) Amino terminal cytosolic region of WT, l-, and s-CD317 constructs. (B) Cell lysates from HeLa cells transiently transfected with the indicated CD317-HA-SR constructs were prepared in sample buffer, separated by SDS-PAGE and immunoblotted with antibodies specific to α-tubulin or HA. Molecular mass markers are indicated in kilodaltons. (C) HeLa cells were transfected with the indicated CD317-HA-SR construct and, 48 hours later, permeabilised by methanol fixation followed by immunofluorescence detection of the HA epitope to visualise whole cell localisation of CD317. (D) HeLa cells were co-transfected with siRNA targeting CD317 and the indicated CD317-HA-SR constructs and, 48 hours later, incubated with an antibody specific to HA to look at protein expression, before surface expression was monitored by flow cytometry in the PE channel. Quantification of CD317-HA-SR surface expression. Values indicate total fluorescence of transfected cells. (E) HEK293-T cells were transfected with the indicated CD317-HA-SR constructs and, 24 hours later, incubated with an anti-HA antibody before surface expression was monitored by flow cytometry in the PE channel. Values indicate total fluorescence of transfected cells. (F,G) DRM/non-DRM localisation of CD317-HA-SR constructs. HeLa (F) or HEK293-T cells (G) transfected with the indicated construct were lysed and DRMs prepared as described in Materials and Methods. DRM localisation (protein in flotillin-2 positive fractions) was quantified by densitometry, with values given as protein localised to DRM as a percentage of total protein. Data represent mean (± s.e.m.) from two independent experiments. Scale bars: 10 µm.

In a recent publication (Cocka and Bates, 2012), expression of two distinct isoforms of CD317 was described for the first time. The two isoforms, designated long (l-CD317) and short (s-CD317), are produced through alternative translation initiation from two methionine residues within the cytosolic domain of CD317. Whilst the l-CD317 isoform is generated via initiation at the methionine residue at position 1, translation initiation at a downstream methionine (at position 13) results in production of the s-CD317 isoform, which is thus twelve amino acids shorter than the l-CD317 isoform (Cocka and Bates, 2012) (Fig. 6A). The two isoforms of CD317 differ significantly in their biological properties. Whilst l-CD317 is sensitive to HIV-1 Vpu-mediated downregulation and potently activates the NF-κB pathway, s-CD317 is highly resistant to HIV-1 Vpu-mediated downregulation, and does not activate NF-κB (Cocka and Bates, 2012). Given these divergent properties, and given also that our data indicate that the full cytosolic domain of CD317 behaves as a ‘membrane microdomain-exclusion’ motif (Figs 3, 5), we reasoned that the two isoforms of CD317 might also differ in their affinity for membrane microdomains, and that this might explain their divergent properties. We therefore generated the two isoforms in the CD317-HA-SR backbone (Fig. 6A). Exclusive generation of l-CD317 was achieved by mutating the M13 residue to an isoleucine (M13I); this mutation has previously been shown to prevent production of the s-CD317 isoform (Cocka and Bates, 2012). To produce an s-CD317 isoform construct, the first twelve amino acids were removed from the cytosolic domain of CD317-HA-SR, resulting in the methionine at position 13 being the site of translation initiation.

Cocka and Bates used HeLa and HEK293-T cells for their studies, in addition to HT1080 cells (Cocka and Bates, 2012). Consequently, we also employed both HeLa and HEK293-T cells. Both l-CD317 and s-CD317 were expressed and delivered to the cell surface with an efficiency that was similar to that of WT CD317-HA-SR in transiently transfected HeLa and HEK293-T cells (Fig. 6B–E; supplementary material Fig. S4). Subsequently, the membrane microdomain localisation of the two isoforms was examined by transient transfection of HeLa or HEK293-T cells followed by lysis in Triton X-100 and separation of samples by ultracentrifugation on sucrose density gradients. The DRM affinity of both the long and short CD317 isoforms was similar to WT CD317-HA-SR, in HeLa and HEK293-T cells (Fig. 6F,G; see supplementary material Fig. S5 for immunoblots). This indicates that the DRM affinity of the two CD317 isoforms is similar, and that the removal of the first twelve amino acids of the cytosolic domain of CD317 does not significantly impact upon the partitioning of CD317 into membrane microdomains.

### The long and short isoforms of CD317 differentially activate the NF-κB pathway despite the fact that both are localised to membrane microdomains with similar efficiency

**Fig. 7.**
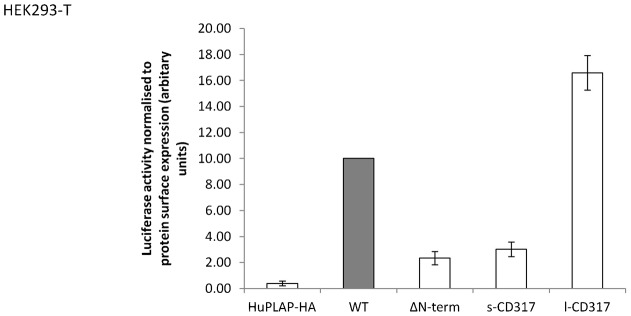
The long and short isoforms of CD317 differentially activate the NF-κB pathway. HEK293-T cells were transfected with reporter plasmids (50 ng of pNF-κB-Luc, 12.5 ng of pRL-SV40) and 50 ng of experimental plasmids encoding WT CD317-HA-SR or, for a negative control, HA-tagged placental alkaline phosphatase (HuPLAP-HA). Twenty-four hours later, cells were lysed, and Firefly and Renilla luciferase activities measured, and then standardised for differences in protein expression by flow cytometry. Flow cytometry on each construct was performed in duplicate 12 well plates contemporaneously with the luciferase experiments, each treatment of which was performed in octuplicate. Data are presented as Firefly:Renilla activity ratios normalised to FACS protein surface expression data. Data represent the mean (± s.e.m.) from two independent experiments.

Membrane microdomains have been implicated in NF-κB signalling (Oakley et al., 2009; Waterfield et al., 2010; Huang et al., 2011), and so a protein's relative affinity for such microdomains could impact upon that protein's capacity to activate NF-κB. Thus, given that the affinity of the long and short isoforms of CD317 for membrane microdomains did not differ significantly from one another, it might have been expected that the two proteins would also activate the NF-κB pathway to a similar degree. However, and consistent with previous observations (Cocka and Bates, 2012), the two isoforms differed markedly in their ability to activate NF-κB (Fig. 7). Whilst the l-CD317 isoform potently activated the NF-κB pathway, to a level almost two-fold greater than that of WT CD317, s-CD317 was a poor activator of the pathway. Indeed, the s-CD317 isoform was only slightly more effective at activating NF-κB than a CD317 construct lacking the entirety of the cytosolic domain (ΔN-term, Fig. 7).

## Discussion

The data presented herein further our understanding of both the association of GPI-anchored proteins generally, and CD317 specifically, with membrane microdomains. The GPI anchor is essential for membrane microdomain association of CD317, and the heterologous expression of the TM domain of CD44 in ΔGPI-CD317 is unable to restore CD317's partitioning into membrane microdomains. For the first time, the relative affinity for membrane microdomains of two recently described isoforms of CD317 is examined, and our data indicate that the two isoforms maintain a similar affinity for membrane microdomains. Furthermore, we present the first evidence, to our knowledge, of a protein ‘membrane microdomain-exclusion’ signal, with the heterologous expression of the cytosolic region of CD317 reducing the membrane microdomain association of Art-Teth. It is of note that whilst addition of the cytosolic tail of CD317 to Art-Teth reduced membrane microdomain association of Art-Teth, its removal from WT CD317 had little effect on the association of the molecule with DRMs. This is consistent with there being a hierarchy of ‘membrane microdomain-association’ and ‘membrane microdomain-exclusion’ motifs/signals in CD317 (and in other proteins) and that, in the case of WT CD317, the N-terminal cytosolic domain is insufficient to over-ride the microdomain-association characteristics, whereas in the case of Art-Teth it is able to do so.

The requirement of the GPI addition motif for membrane microdomain partitioning of CD317 in HEK293-T cells, as illustrated herein (Fig. 1) is entirely consistent with our previous findings in HeLa cells (Billcliff et al., 2013). In addition, other research has shown that the GPI anchor is required for CD317 to associate with DRMs (Kupzig et al., 2003; Masuyama et al., 2009; Rollason et al., 2009). Furthermore, the GPI anchor is required for clustering of CD317 molecules on the cell surface (Lehmann et al., 2011). Although our data suggest an absolute requirement for the GPI anchor for membrane microdomain association of CD317, with the TM domains of neither CD8 nor CD44 effectively restoring membrane microdomain association of CD317-HA-SR ΔGPI (Fig. 1) (Billcliff et al., 2013), contradictory data are provided in the work of Andrew et al. (Andrew et al., 2011). In this paper (Andrew et al., 2011), the group found that the addition of the TM domain of CD40 – which has previously been shown to be required for membrane microdomain localisation of CD40 (Vidalain et al., 2000; Bock and Gulbins, 2003) – was sufficient to partition the resultant ΔGPI-CD40 chimera into membrane microdomains (Andrew et al., 2011). More surprisingly, two other chimeric constructs, ΔGPI-CD45 and ΔGPI-TfR, containing the TM domains of the non-membrane microdomain proteins CD45 (Cheng et al., 1999) and the TfR (Sakane et al., 2010), respectively, also localized to membrane microdomains with similar efficiency to the ΔGPI-CD40 protein (Andrew et al., 2011). In sharp contrast, the ΔGPI-CD8 construct used here, containing the non-membrane microdomain TM domain of CD8α (Pang et al., 2007), is excluded from DRMs. It is hard to reconcile the contrasting membrane microdomain affinities of the CD317 ΔGPI chimeric constructs we have generated and those described by Andrew et al. However, given that the ΔGPI-CD44 and ΔGPI-CD44P constructs we have generated are not efficiently palmitoylated in HEK293-T cells (Fig. 4), it is conceivable that improper post-translational modification of these constructs may underlie their impaired partitioning into membrane microdomains, relative to both WT CD317-HA-SR (Fig. 1) (Billcliff et al., 2013) and the ΔGPI-CD40 construct employed in the work of Andrew et al. (Andrew et al., 2011). Based on their findings, including data that suggested that the C-terminal region of CD317 protrudes into the cytoplasm, Andrew et al. concluded that human CD317 contains a second TM domain rather than a GPI addition sequence. However, it is of note that proteomics analysis has independently identified CD317 as a GPI-anchored protein (Elortza et al., 2006). Thus, it is possible that CD317 can exist with either a C-terminal GPI anchor or a C-terminal TM domain.

The s-CD317 isoform, lacking the first twelve amino acids at the amino terminus of the protein, is impaired in its ability to activate the NF-κB pathway, but also more resistant to HIV-1 Vpu-mediated downregulation, relative to the l-CD317 isoform (Cocka and Bates, 2012). However, the relative affinities of the two isoforms for membrane microdomains are similar in both HeLa and HEK293-T cells (Fig. 6). This suggests that the first twelve amino acids of CD317 are not important for membrane microdomain association of the protein. Nevertheless, the cytosolic region, as a whole, functions in determining protein membrane microdomain association, given that the inclusion of the cytosolic tail of CD317 in a heterologous protein, Art-Teth, reduces the resultant protein's affinity for membrane microdomains (Fig. 5). Consistent with the N-terminal domain of CD317 behaving as a ‘membrane microdomain-exclusion’ signal, the removal of this region from the ΔGPI-CD44 CD317-HA-SR construct leads to an increase in membrane microdomain association of that protein in HEK293-T cells (Fig. 3). The observation that the cytosolic region of a protein can act to restrict or exclude a normally membrane microdomain-associated protein, is certainly worthy of further study.

## Materials and Methods

### CD317 expression construct and plasmids

The previously described (Rollason et al., 2009) hairpin siRNA oligonucleotide 5′-CCAGGTCTTAAGCGTGAGA-3′ (corresponding to base pairs 432–450 of the human CD317 sequence) was used to knockdown CD317. The creation of WT, ΔN-term, ΔGPI, ΔGPI-CD8, ΔGPI-CD44 and C3A constructs in the siRNA-resistant, HA-tagged CD317 background was described previously (Billcliff et al., 2013). The ΔGPI-CD44P construct was generated in an identical manner to the ΔGPI-CD44 construct, using complementary overlapping primers, except that residues 266 to 300 (rather than 266 to 290) of CD44 transcript variant 4 were incorporated into the chimeric construct. This added a second palmitoylation site, C295, to the resultant protein. The ΔNt-term-ΔGPI-CD44 and ΔNt-term-ΔGPI-CD44P constructs, in which the sequence encoding the first twenty amino acids of CD317 was deleted from the protein, were generated using pcr3.1-ΔGPI-CD44 and pcr3.1-ΔGPI-CD44P plasmids as templates, respectively, and PCR primers to the relevant sequences.

The ‘artificial tetherin’ construct, consisting of part of the transferrin receptor (TfR) cytosolic domain, its TM region and part of the extracellular stalk, the coiled-coil of DMPK (dystrophia myotonica protein kinase), an HA epitope tag and the C-terminus (including GPI addition motif) of uPAR (urokinase plasminogen activator receptor) ligated together to form a protein that mimics tetherin's topology, has been described previously (Perez-Caballero et al., 2009), and was a gift from Paul Bieniasz (Rockefeller University, New York, NY, USA). A chimeric human CD317 artificial tetherin construct, designated WT N-ArtTeth, consisting of the amino terminal cytosolic region of human CD317 attached to the amino terminus of artificial tetherin, was generated by annealing a DNA sequence encoding the entirety of the human CD317 cytosolic region (amino acid residues 1–20) to an ‘artificial tetherin’ PCR product designed to begin at the transmembrane domain of the TfR (therefore lacking the twenty cytosolic amino acids of the TfR found in the original artificial tetherin construct) using complementary overlapping primers.

### Imaging

Immunofluorescence confocal microscopy was performed as described previously (Billcliff et al., 2013). Briefly, HeLa cells grown on cover slips were co-transfected with CD317 siRNA and CD317-HA-siRNA resistant expression constructs (X-tremeGENE; Roche) and cultured for a further 48 hours before processing for immunofluorescence analysis. Cells were then immediately fixed and permeabilised in methanol to allow detection of whole-cell distribution of protein, incubated with the primary anti-HA antibody (Covance) for 1 hr, washed with PBS and then incubated with Alexa-Fluor 488-conjugated secondary donkey anti-mouse antibody for 1 hr. To assay protein delivery to the cell surface, cells were instead incubated with the primary anti-HA antibody for 20 minutes on ice prior to fixation in 3% formaldehyde, washed with PBS, and incubated with the same Alexa-Fluor 488-conjugated secondary antibody for 1 hr. Labelled cells were imaged using a confocal laser-scanning microscope (AOBS SP2; Leica) equipped with Ar (458, 476, 488, 496, 514 nm lines) and 405 nm diode lasers attached to an inverted epifluorescence microscope (DMRBE2; Leica). Images were collected using a 63× NA 1.4 oil immersion objective and processed with Leica and Photoshop (Adobe) software.

### Detergent resistant membrane isolation

Cells were grown in 10 cm plates to 50% confluency, transfected with siRNA and/or plasmid and incubated for a further 48 hours. On ice, cells were scraped into 2 mls of TNE + 1% Triton X-100 and passed 8 times through a 21 g needle. After 30 mins incubation on ice the lysate was bought up to 40% sucrose by addition of 2 mls of 80% sucrose in TNE in a 12 ml centrifuge tube. Five mls of 35% sucrose in TNE was layered on top followed by 1 ml each of 15% sucrose, 1% sucrose and TNE. The gradients were spun at 34 000 rpm in a Sorval TH.641 swing out rotor for 18 hours at 4°C. One ml fractions were taken and the protein precipitated by addition of 0.25 volume of 100% TCA (Trichloroacetic Acid). Fractions were resuspended in sample buffer (10% sodiumdodecyl sulphate, 10% β-mercaptoethanol).

Protein localisation to DRMs was quantified using Photoshop CS2 (Adobe). Immunoblots were converted to gray-scale images and inverted. Protein bands were selected using the lasso tool copied to a new layer, and mean pixel intensity determined for both total protein (protein from all eleven fractions of the gradient) and for DRM-localised protein (protein in the flotillin-2-positive fractions). Excel 2007 (Microsoft) was used to generate bar charts, representing the percentage of total protein localised to DRMs.

### Luciferase reporter assay

Luciferase assays were performed in 96 well plates as described previously (Billcliff et al., 2013). Briefly, in each well of a black 96-well plate (Greiner), 1×10^4^ 293-T cells were seeded and, 24 hours later, transfected with 50 ng of CD317 or control plasmid together with 50 ng of reporter plasmid and 12.5 ng of transfection control plasmid, using 0.4 µl Genejuice (Merck Chemicals); total DNA levels were equalised with sheared salmon sperm DNA (Sigma). Twenty-four hours post-transfection, cells were harvested and assayed using the Dual-Glo Luciferase System (Promega), according to the manufacturer's instructions. The reporter plasmid, pNF-κB-Luc, contains Firefly luciferase downstream of an NF-κB responsive promoter; the transfection control plasmid, pRL-SV40 (Promega), contains Renilla luciferase downstream of the constitutive SV40 promoter. Negative and positive controls were performed using pGL3, where Firefly luciferase is under the control of no promoter, and pFC-MEKK (Stratagene), respectively. Each treatment was carried out in octuplicate.

To take protein expression variations into account, flow cytometry was performed contemporaneously with the luciferase assay. In each well of a 12 well plate, 1.27×10^5^ 293-T cells were seeded and, 24 hours later, transfected with the same mixture of plasmids as were the 96 well plates, except that each well of a 12 well plate was treated with 12.7 times the amount of transfection mixture used for a well of a 96 well plate. Twenty-four hours post-transfection, cells were washed in PBS and resuspended in PBSA (PBS, 1% BSA) containing primary anti-HA antibody, and incubated for 1 hour. Cells were then washed once in ice-cold PBS, and incubated with PE conjugated anti-mouse secondary antibodies for 1 hour at 4°C. Fluorescence signals were measured using a FACS CantoII-F60 machine (BD Biosciences, Oxford, UK). Data were analyzed using Flowjo 7.2.5 software (Flowjo, Ashland, OR, USA). Each treatment was performed in duplicate. Subsequent to data analysis, luciferase data were normalised to mean PE fluorescence signals.

## Supplementary Material

Supplementary Material
