# Supplementary material for: The cytosolic N-terminus of CD317/tetherin is a membrane microdomain exclusion motif
Source: Biol Open. 2013 Oct 15;2(11):1253–63. doi: 10.1242/bio.20135793 (PMC3828773; doi:10.1242/bio.20135793)
Supplement: Supplementary Material [file supp_2_11_1253__index.html]

The cytosolic N-terminus of CD317/tetherin is a membrane microdomain exclusion motif — Supplementary Material 

# The cytosolic N-terminus of CD317/tetherin is a membrane microdomain exclusion motif

## bio.20135793 Supplementary Material

**Files in this Data Supplement:**

- Supplementary Material - Peter G. Billcliff et al. doi: 10.1242/bio.20135793
