# Supplementary material for: The cytosolic N-terminus of CD317/tetherin is a membrane microdomain exclusion motif
Source: Biol Open. 2013 Oct 15;2(11):1253–63. doi: 10.1242/bio.20135793 (PMC3828773; doi:10.1242/bio.20135793)
Supplement: Supplementary Material [file supp_bio.20135793_bio.20135793-s1.pdf]

Biology Open

Supplementary Material

Peter G. Billcliff et al. doi: 10.1242/bio.20135793

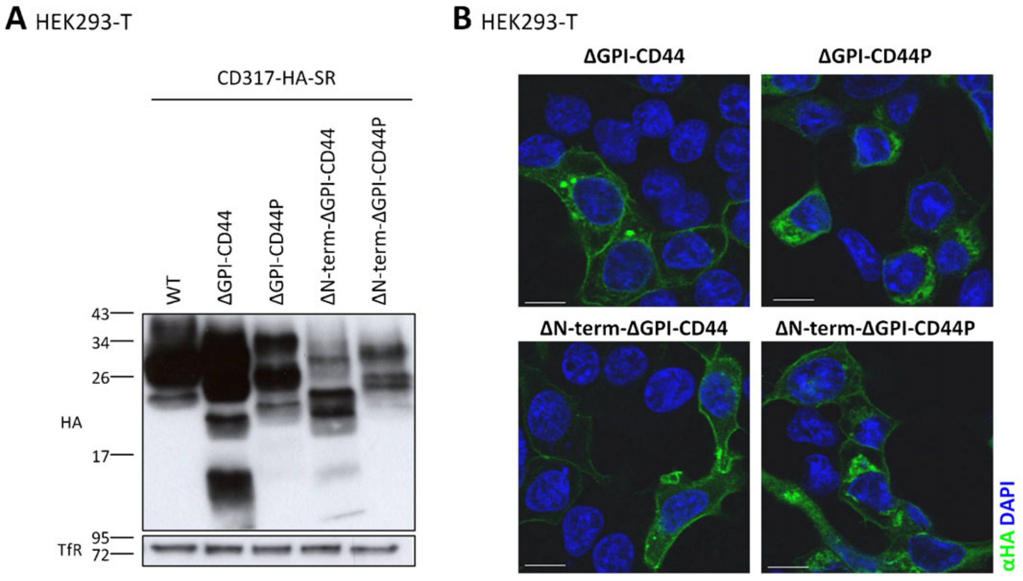

Fig. S1. Expression of CD317-ΔGPI-CD44 constructs in HEK293-T cells. (A) Cell lysates from HEK293-T cells transiently transfected with the indicated CD317-HA-SR constructs were prepared in sample buffer, separated by SDS-PAGE and immunoblotted with antibodies specific to HA or the TfR. Molecular mass markers are indicated in kilodaltons. (B) HEK293-T cells were transiently transfected with the indicated CD317-HA-SR constructs and, 24 hours later, permeabilised by methanol fixation followed by immunofluorescence detection of the HA epitope to visualise whole cell localisation of CD317. Scale bars: 10 μm.

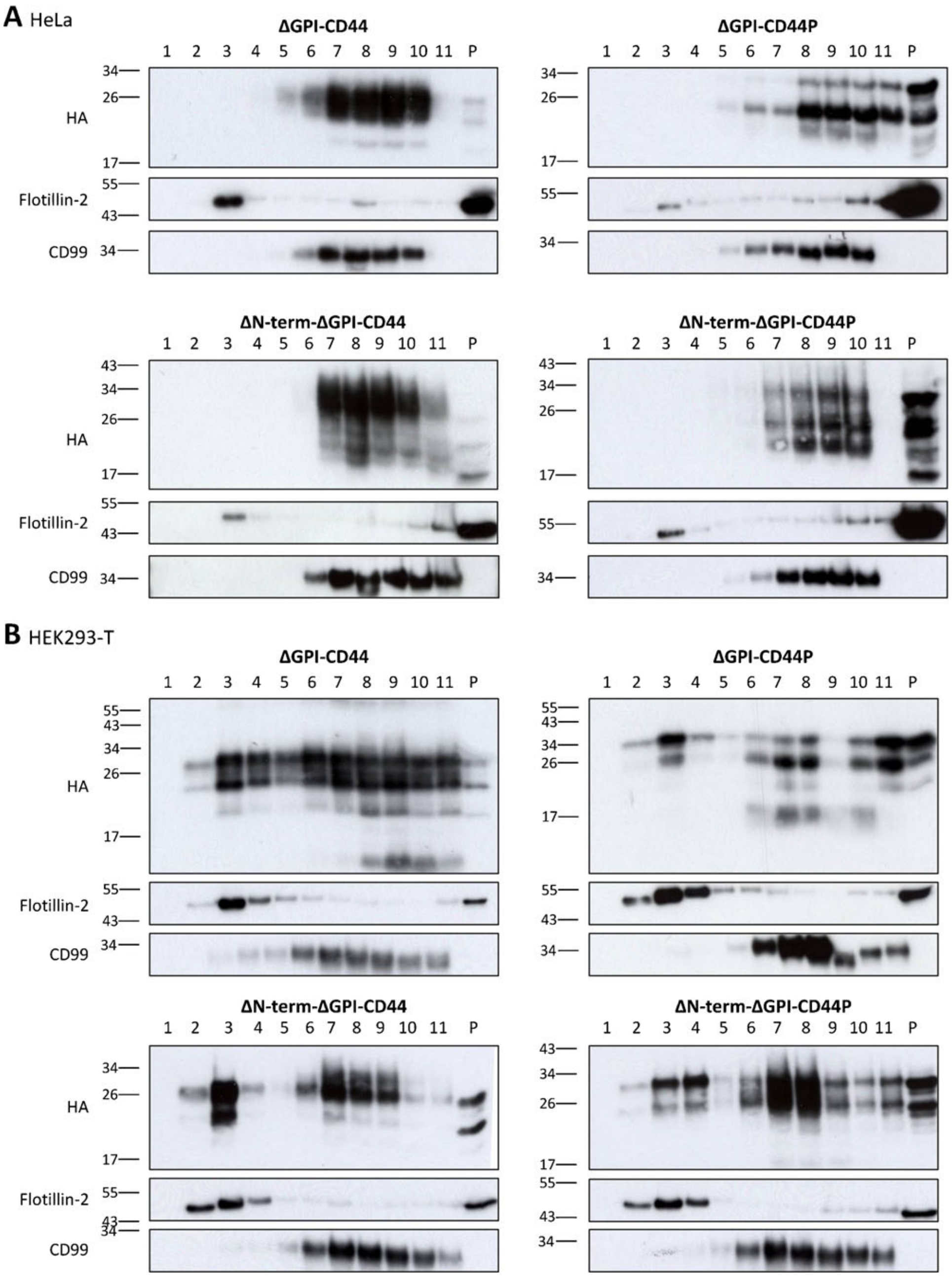

Fig. S2. DRM/non-DRM localisation of CD317-ΔGPI-CD44 constructs. Immunoblot analysis of fractions from sucrose-density-gradient separation of HeLa (A) or HEK293-T (B) cell lysates from cells transfected with the indicated CD317 constructs. Fractions were taken from the top of the gradient (i.e. fraction 1 is the most buoyant), and blots were probed with HA, flotillin-2 and CD99 antibodies, as indicated.

Biology Open

Biology Open

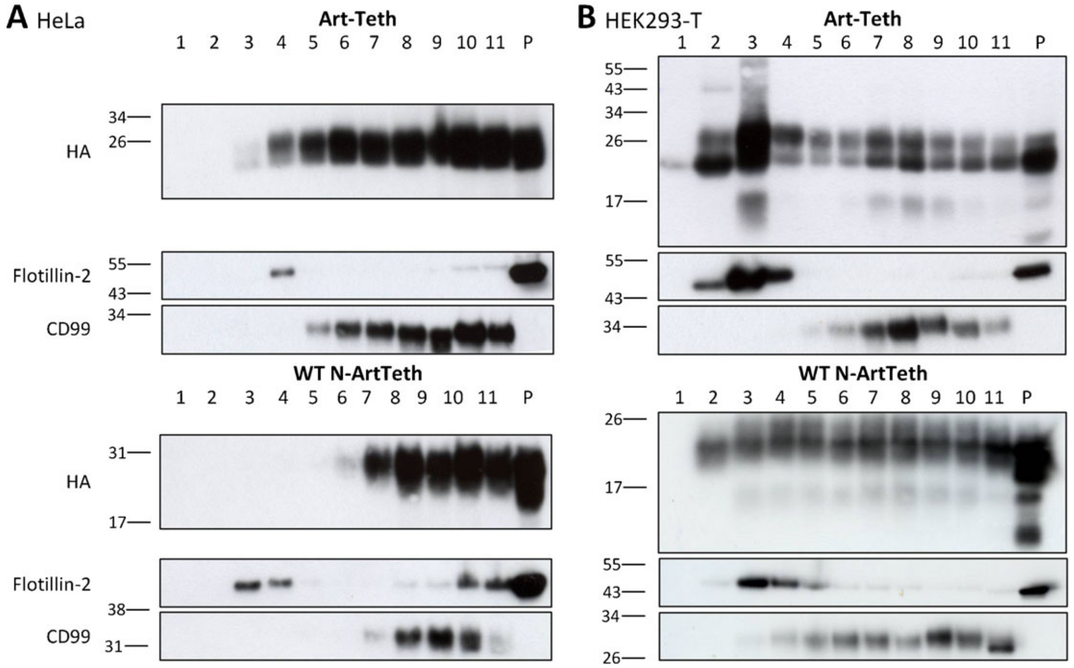

Fig. S3. DRM/non-DRM localisation of artificial tetherin constructs. Immunoblot analysis of fractions from sucrose-density-gradient separation of HeLa (A) or HEK293-T (B) cell lysates from cells transfected with the indicated tetherin constructs. Fractions were taken from the top of the gradient (i.e. fraction 1 is the most buoyant), and blots were probed with HA, flotillin-2 and CD99 antibodies, as indicated.

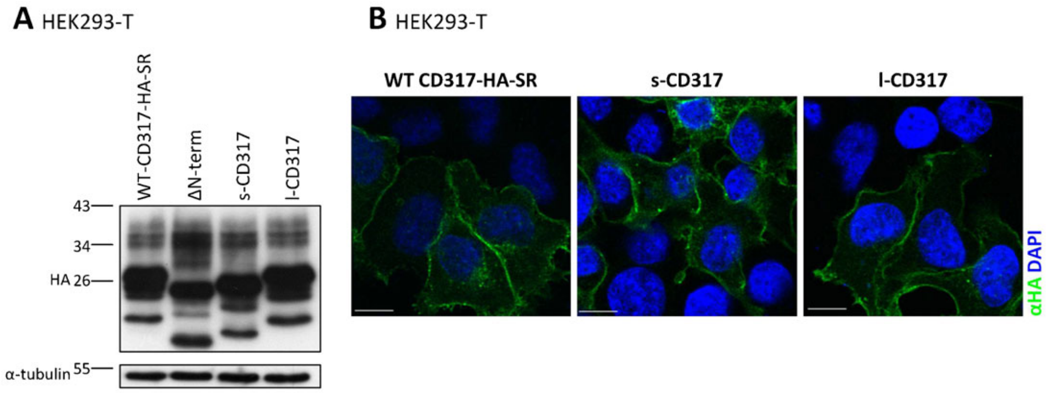

Fig. S4. Expression of l- and s-CD317 isoform constructs in HEK293-T cells. (A) Cell lysates from HEK293-T cells transiently transfected with the indicated CD317-HA-SR constructs were prepared in sample buffer, separated by SDS-PAGE and immunoblotted with antibodies specific to HA or the TfR. Molecular mass markers are indicated in kilodaltons. (B) HEK293-T cells were transiently transfected with the indicated CD317-HA-SR constructs and, 24 hours later, permeabilised by methanol fixation followed by immunofluorescence detection of the HA epitope to visualise whole cell localisation of CD317. Scale bars: 10 µm.

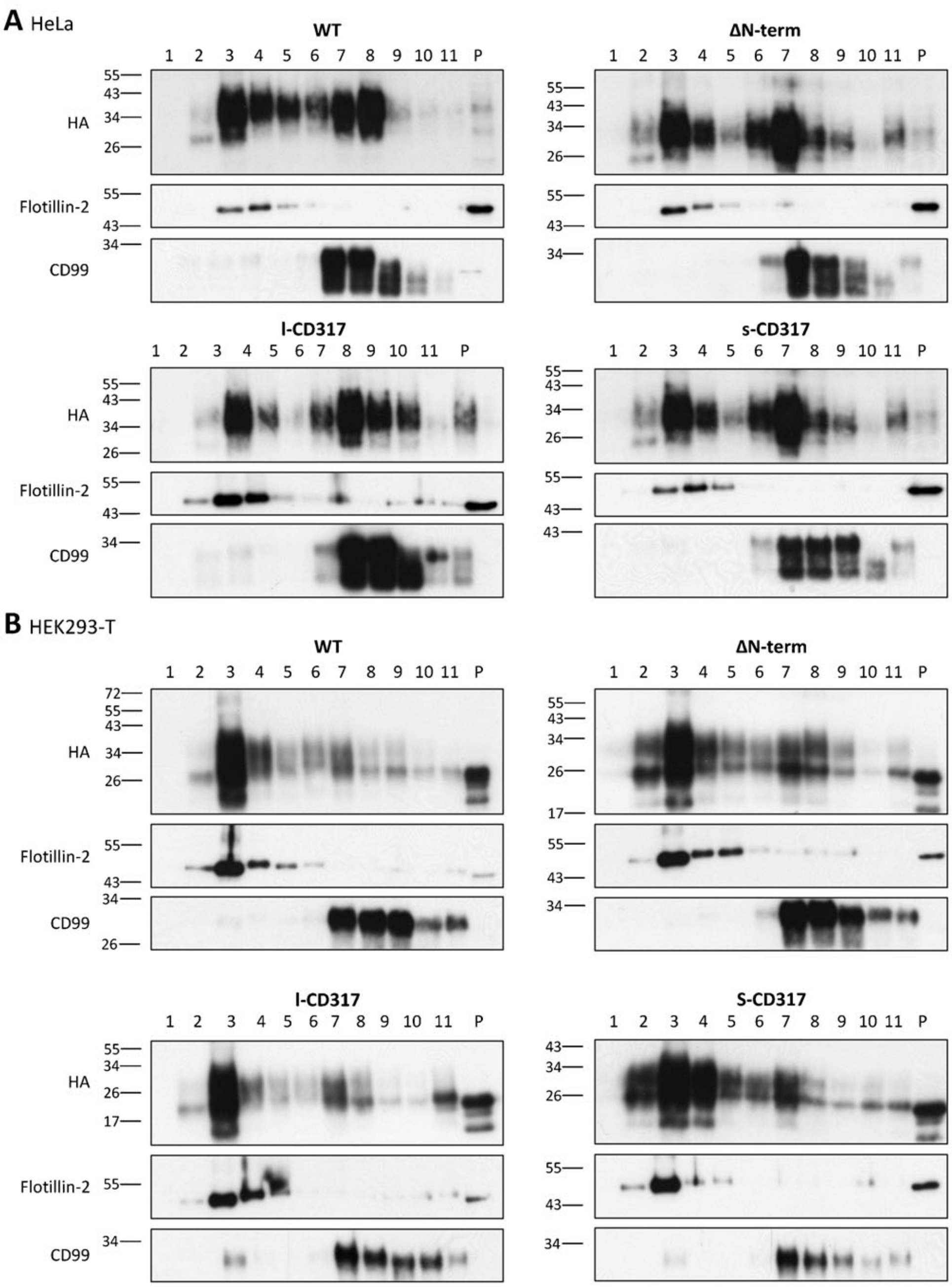

Fig. S5. DRM/non-DRM localisation of l- and s-CD317 isoforms. Immunoblot analysis of fractions from sucrose-density-gradient separation of HeLa (A) or HEK293-T (B) cell lysates from cells transfected with the indicated CD317 constructs. Fractions were taken from the top of the gradient (i.e. fraction 1 is the most buoyant), and blots were probed with HA, flotillin-2 and CD99 antibodies, as indicated.
